# Associations of chest X-ray trajectories, smoking, and the risk of lung cancer in two population-based cohort studies

**DOI:** 10.3389/fonc.2023.1203320

**Published:** 2023-07-18

**Authors:** Ya Liu, Zhuowei Feng, Zeyu Fan, Yu Zhang, Chenyang Li, Xiaomin Liu, Hongyuan Duan, Xiaonan Cui, Liwen Zhang, Chao Sheng, Lei Yang, Ying Gao, Xing Wang, Qing Zhang, Zhangyan Lyu, Fangfang Song, Yubei Huang, Fengju Song

**Affiliations:** ^1^ Department of Epidemiology and Biostatistics, National Clinical Research Center for Cancer, Key Laboratory of Molecular Cancer Epidemiology of Tianjin, Key Laboratory of Cancer Prevention and Therapy of Tianjin, Tianjin’s Clinical Research Center for Cancer, Tianjin Medical University Cancer Institute and Hospital, Tianjin Medical University, Tianjin, China; ^2^ Department of Radiology, National Clinical Research Centre for Cancer, Key Laboratory of Cancer Prevention and Therapy of Tianjin, Tianjin Medical University Cancer Institute and Hospital, Tianjin, China; ^3^ Hebei Key Laboratory of Environment and Human Health, Department of Epidemiology and Statistics, School of Public Health, Hebei Medical University, Shijiazhuang, China; ^4^ Key Laboratory of Carcinogenesis and Translational Research (Ministry of Education), Beijing Office for Cancer Prevention and Control, Peking University Cancer Hospital and Institute, Beijing, China; ^5^ Health Management Center, Tianjin Medical University General Hospital, Tianjin, China

**Keywords:** lung cancer, chest X-ray, trajectory, smoking, incidence, mortality

## Abstract

**Objectives:**

Despite the increasing use of computed tomography (CT), chest X-ray (CXR) remains the first-line investigation for suspected lung cancer (LC) in primary care. However, the associations of CXR trajectories, smoking and LC risk remain unknown.

**Methods:**

A total of 52,486 participants from the PLCO and 22,194 participants from the NLST were included. The associations of CXR trajectories with LC risk were evaluated with multivariable COX regression models and pooled with meta-analyses. Further analyses were conducted to explore the stratified associations by smoking status and the factors associated with progression and regression in CXR.

**Results:**

Compared to stable negative CXR (CXR_SN_), HRs (95%CIs) of LC incidence were 2.88(1.50–5.52), 3.86(2.03–7.35), and 1.08(0.80–1.46) for gain of positive CXR (CXR_GP_), stable positive CXR (CXR_SP_), and loss of positive CXR (CXR_LP_), while the risk of LC mortality were 1.58(1.33–1.87), 2.56(1.53–4.29), and 1.05(0.89–1.25). Similar trends were observed across different smoking status. However, LC risk with CXR_GP_ overweighed that with CXR_SP_ among ever smokers [2.95(2.25–3.88) *vs*. 2.59(1.33–5.02)] and current smokers [2.33(1.70–3.18) *vs*. 2.26(1.06–4.83)]. Moreover, compared to CXR_SN_ among never smokers, even no progression in CXR, the HRs(95%CIs) of LC incidence were 7.39(5.60–9.75) and 31.45(23.58–41.95) for ever and current smokers, while risks of LC mortality were 6.30(5.07–7.81) and 27.17(21.65–34.11). If participants gained positive CXR, LC incidence risk significantly climbed to 22.04(15.37–31.60) and 71.97(48.82–106.09) for ever and current smokers, while LC mortality risk climbed to 11.90(8.58–16.50) and 38.92(27.04–56.02). CXR_LP_ was associated with decreased LC risk. However, even smokers lost their positive CXR, and the increased risks of LC incidence and mortality did not decrease to non-significant level. Additionally, smoking was significantly associated with increased risk of CXR_GP_ but not CXR_LP_.

**Conclusion:**

LC risk differed across CXR trajectories and would be modified by smoking status. Comprehensive intervention incorporating CXR trajectories and smoking status should be recommended to reduce LC risk.

## 1 Introduction

Lung cancer (LC) ranks as the most common cancer and the leading cause of cancer mortality in men for several years, while it is the third common cancer and the second leading cause of mortality in women in 2020 around the world (1–3). In 2020, an estimated 2.2 million new LC cases and 1.8 million LC deaths occurred, which accounted for 11.4% of all new cancer cases and 18.0% of all cancer deaths (4). Due to the emerging aging trend, stubbornly high tobacco epidemic, and surging air pollution, the LC incidence is expected to continually rise in many countries in the future. Reducing the increasing burden of LC has become a global concern faced by several countries, especially by transitioning or developing countries.

Although low-dose computed tomography (LDCT) has greatly altered the landscape of LC screening since 2011 (5, 6), chest X-ray (CXR) is still the first-line examination of lung cancer in primary healthcare due to more universal availability (7), less radiation dose (8), less requirement for technicians, and relatively lower cost than LDCT. Moreover, with the widespread rise in artificial intelligence (AI), deep-learning-based automatic diagnostic model based on CXR is highly expected to significantly improve the early detection rate of LC (9–11). However, before the sophisticated AI-assisted CXR diagnostic technology is widely used in resource-limited regions, how to reduce the potential missed diagnosis and false positive diagnosis associated with traditional CXR exam is the key to improve the effect of CXR examination. Currently, the associations of different CXR trajectories and LC risk remain unknown. Ignoring the CXR trajectories, especially the progression and regression in CXR, is presumed to be the leading causes of the non-significant reduction in LC mortality for previous CXR screening trials. Independent evaluation of CXR without referring to other risk factors (especially for smoking) may also dilute the effects of CXR screening for LC. However, until now, few studies have investigated the associations of CXR trajectories with LC risk, and no study has explored the stratified effects by smoking and the interaction between CXR and smoking on LC risk.

Therefore, in this study, based on the analysis for secondary data from the Prostate, Lung, Colorectal, and Ovarian Cancer (PLCO) Screening (12–15) trial and the National Lung Screening Trial (NLST) (6), we first aimed to investigate the associations of CXR trajectories with LC incidence and mortality, and meta-analyses were conducted to achieve the pooled results beyond the individual study. Furthermore, we aimed to evaluate the stratified associations by smoking status, the interaction between CXR and smoking on LC risk, and the potential factors associated with progression and regression in CXR.

## 2 Material and methods

### 2.1 Source of population

The trial registration number (on ClinicalTrials.gov) of PLCO is NCT00002540, and the trial registration number (on ClinicalTrials.gov) of NLST is NCT00047385. The designs of the PLCO and NLST cancer screening trials have been previously published. Briefly, from 1993 to 2001, the PLCO cancer screening trial randomized 154,887 participants aged 55–74 years to the intervention arm receiving multiple screening exams for prostate, lung, colorectal, and ovarian cancers or to the control arm receiving usual care in 1:1 ratio (12–15). For the LC screening, participants in the screening arm received four annual posterior–anterior CXR. There were several changes in the screening protocol, including that never smokers randomized after December 1995 were no longer offered a T3 CXR exam unless they insisted on it. Positive screening exam of CXR was defined as one or more nodules, mass, hilar or mediastinal lymph node enlargement, infiltrate, consolidation, or alveolar opacity. Participants with positive CXR were encouraged to receive further diagnostic evaluation with their primary care physicians. From 2002 to 2004, the NLST cancer screening trial randomized 53,452 smokers aged 55–74 years with at least 30 pack-years of smoking history and at most 15 years smoking cessation history to receive three annual LDCT screening (the intervention arm) or posterior–anterior CXR screening (the control arm) in 1:1 ratio (6). Radiologists were required to review the images in two ways: looking at the image without reference to historical images (isolation read) and then looking at the image again with reference to historical images (comparison read). Positive exam was defined as any non-calcified nodule or mass with diameter ≥ 4 mm, or any other abnormalities appeared suspicious for lung cancer (in the radiologist’s judgment). Participants with either a positive result or a negative result but with other clinically significant abnormalities were strongly encouraged to receive a diagnostic evaluation for lung cancer or other suspected condition. Each participating center’s institutional review board approved the protocols, and all participants provided written informed consent.

### 2.2 Selection of participants

In the PLCO trial, after excluding the participants in the control arm, a total of 77,443 participants in the screening arm were initially included in this study. After further excluding 6,811 participants who did not receive any CXR examination or without inadequate screen, 3,819 participants with one round of CXR examination, 4,937 participants with two rounds of CXR examination, 2,620 participants with three rounds of CXR examination enrolled in 1993–1995, 3,175 smoking participants with three rounds of CXR examination enrolled in 1996–2001, and 3,595 participants who did not meet the definitions of four classic CXR trajectories (details referred to the following section) in CXR, a total of 52,486 participants were finally included in this study (**Supplementary e-Figure S1**). In the NLST trial, after excluding the participants who received LDCT in the intervention arm, a total of 26,730 participants who received CXR in the control arm were initially included in this study. After further excluding 4,037 participants without any CXR examinations and 699 participants who did not meet the definitions of four classic CXR trajectories (details referred to the following section) in CXR, a total of 21,994 participants were finally included in this study (**Supplementary e-Figure S2**).

### 2.3 Determination of CXR trajectories

Based on multiple rounds of CXR screening, to achieve comparable CXR trajectories between the PLCO and NLST trials, four classic CXR trajectories were defined in this study, including stable negative CXR (CXR_SN_), gain of positive CXR after persistent negative CXR and no subsequent negative diagnosis (CXR_GP_), stable positive CXR (CXR_SP_), and loss of positive CXR after persistent positive CXR and no subsequent positive diagnosis (CXR_LP_). To avoid the confounding effect of instability symptoms on four classic CXR trajectories, disordered fluctuations on CXR were not included in this study, including that negative CXR progressed to positive CXR and subsequently regressed to negative CXR again, and that positive CXR regressed to negative CXR and subsequently progressed to positive CXR again.

### 2.4 Information of baseline variables

After informed consent, all participants in the PLCO trial were provided with a baseline questionnaire to collect participant-reported information on demographic and potential risk factors associated with PLCO cancers, such as demographics, smoking history, family history of cancer, and medical history. Similar baseline variables with different information were collected in the NLST trial. To achieve comparable data in both the PLCO and NLST trials, the following index variables were finally included in this study, including age (55–59, 60–64, 65–69, and 70–74 years), sex (female and male), race/ethnicity (non-Hispanic white and other), education level (<senior high school, senior high school, college, and above), marital status (married/living as married, widowed/divorced/separated, and never married), smoking status (never smoking [only for the PLCO trial], ever smoking, and current smoking), body mass index (BMI) (<18.5, 18.5–25, 25–30, and > 30 kg/m^2^), and family history of lung cancer (no and yes). BMI was calculated as weight in kilograms divided by the square of height in meters (kg/m^2^).

### 2.5 Ascertainment of endpoints

The primary endpoints events of this study were LC incidence and mortality. In the PLCO trial, the incidence and mortality of LC were ascertained primarily by mails of Annual Study Update (ASU) questionnaire after last-round CXR and supplemented by repeated mails or phone calls to participants who were not response to the ASU questionnaire. The mortality was further supplemented by periodic linkage to the National Death Index (NDI), and a more accurate assessment of LC deaths was adjudicated by an independent Death Review Process (DRP). The cancer data were collected until 31 December 2009, and mortality data were collected through 2018 in the PLCO trial.

The NLST confirmed diagnoses of LC through medical record abstraction (MRA), which was triggered by annual or semi-annual study update form, positive CT or CXR screening exam, direct report by relatives or physicians, and supplemented by NDI Plus searches. If the MRA process did not find records indicating a LC diagnosis, the LC was not considered confirmed, even if a source such as a death certificate indicated LC. Endpoint verification process was used to determine definitively whether LC was the cause of death. Active follow-up data were collected on cancer diagnoses and deaths that occurred through 31 December 2009. Extended follow-up data were collected for deaths through 31 December 2015.

In both the PLCO and NLST trials, if LC was diagnosed, further information was recorded about cancer characteristics (including histopathological type, grade, location, size of tumor, and TNM components of stage), initial treatment, and cancer progression. Furthermore, in this study, the primary outcomes in both the PLCO and NLST trials were censored at the date of the LC diagnosis (for LC incidence only), death, loss of follow-up, or end of the follow-up period, whichever comes first.

### 2.6 Statistical analysis

In this analysis for secondary data, analysis of variance or chi-square test was performed to compare the distribution of baseline variables and pathological characteristics of LC between different groups. Log-rank test was initially used to compare the LC incidence and mortality between four classic CXR trajectories. Multivariable Cox proportional hazard regression model was used to analyze the associations of CXR trajectories with LC incidence and mortality after adjusting all available baseline variables (including age, sex, race, education levels, marital status, smoking status, family history of lung cancer, and BMI). The associations were measured as hazard ratio (HR) and 95% confidence interval (CI). Due to the potential heterogeneity between the PLCO and NLST trials, meta-analyses with random-effect models were conducted to pool the study-specific associations from the PLCO and NLST trials.

Subgroup analyses were performed to evaluate the stratified associations of CXR trajectories with LC risks by smoking status in the PLCO trial but not the NLST trial due to lack of non-smokers in the NLST trial. Further analyses were conducted to interaction between CXR trajectories and smoking with LC risk in the PLCO trial. Additionally, multivariable logistic regression models were used to investigate the potential factors associated with progression and regression in CXR, and associations were measured as odd ratio (OR) and 95% confidence interval (CI).

All statistical analyses were conducted via R software (version 4.1.0). A p-value < 0.05 was considered statistically significant.

## 3 Results

### 3.1 Baseline characteristics between different CXR trajectories

As presented in **Supplementary e-Table S1**, in the PLCO trial, compared to participants with CXR_SN_, those with CXR_GP_ seemed to have older age (70–74 years, 15.7% *vs*. 11.5%, p-value < 0.001), more men (53.1% *vs*. 50.7%, p-value = 0.015), lesser white Americans (88.7% *vs*. 89.9%, p-value = 0.047), lower education level (< senior high school, 7.7% *vs*. 6.2%, p-value = 0.001), lesser married status (75.2% *vs*. 78.7%, p-value < 0.001), and more current smoker (12.7% *vs*. 8.3%, p-value <0.001), while there was no significant difference in baseline characteristics between participants with CXR_SP_ and CXR_LP_. In **Supplementary e-Table S2**, in the NLST trial, only older age was observed in participants with CXR_GP_ than those with CXR_SN_ (70–74 years, 11.0% *vs*. 8.1%, p-value < 0.001), while younger age (70–74 years, 11.4% *vs*. 14.2%, p-value =0.011), more white Americans (92.2% *vs*. 87.6%, p-value = 0.047), and higher BMI (>30 kg/m^2^, 25.3% *vs*. 14.8%, p-value = 0.003) was observed in participants with CXR_LP_ than those with CXR_SP_.

### 3.2 Association of CXR trajectories with LC risk

**Figure 1:**
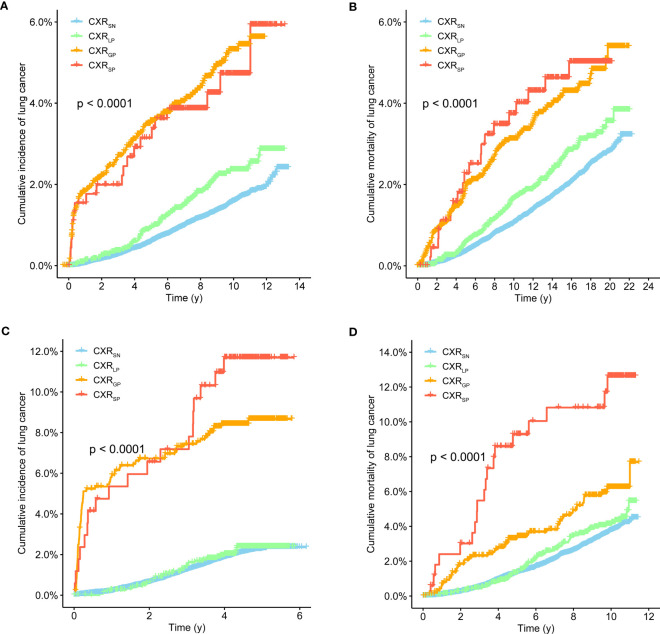
Kaplan–Meier survival curves of lung cancer incidence **(A, C)** and mortality **(B, D)** with CXR trajectories in the PLCO **(A, B)** and NLST **(C, D)** trials.

After a median follow-up of 16.95 and 10.30 years in the PLCO and NLST trials, a total of 889 (1.7%) LC cases and 1,186 (2.3%) LC deaths were documented in the PLCO trial, while a total of 532 (2.4%) LC cases and 823 (3.7%) LC deaths were recorded in the NLST trial. As shown in **Figure 1**, participants with CXR_SP_ seemed to have the higher risks of LC incidence and mortality than participants with other CXP trajectories in the PLCO trial (all p-values < 0.01), and almost the same results were also observed in the NLST trial.

As shown in **Table 1**, after adjusting all available baseline factors, compared to CXR_SN_, CXR_GP_ was significantly associated with increased LC incidence [HR (95%CIs) of 2.82(2.32–3.42) for PLCO, 4.03(3.08–5.28) for NLST, and 3.33(2.35–4.71) for meta-analysis] and LC mortality [1.63(1.33–2.00) for PLCO, 1.45(1.05–2.01) for NLST, and 1.68(1.41–2.01) for meta-analysis]. CXR_LP_ was significantly associated with decreased LC incidence [0.43(0.26–0.71) for PLCO, 0.16(0.09–0.29) for NLST, and 0.27(0.10–0.70) for meta-analysis results] and LC mortality [0.55(0.34–0.88) for PLCO, 0.29(0.17–0.50) for NLST, and 0.40(0.22–0.76) for meta-analysis results] compared to CXR_SP_.

**Table 1:** Association of CXR trajectories with lung cancer incidence and mortality in the PLCO and NLST trials. .

CXR trajectories	Lung cancer incidence			Lung cancer mortality		
	Events/total (%)	Model 1 ^a^ HR (95% CI)	Model 2 ^b^ HR (95%CI)	Events/total (%)	Model 1 ^a^ HR (95% CI)	Model 2 ^b^ HR (95%CI)
PLCO						
First-round negative CXR						
CXR_SN_	669/45,828 (1.5)	1 (ref)	1 (ref)	964/45,828 (2.1)	1 (ref)	1 (ref)
CXR_GP_	123/2,581 (4.8)	2.82 (2.32–3.42)	2.08 (2.31–3.40)	105/2,581 (4.1)	1.63 (1.33–2.00)	1.63 (1.33–2.00)
First-round positive CXR						
CXR_SP_	20/455 (4.4)	1 (ref)	2.79 (1.79–4.36)	21/455 (4.6)	1 (ref)	1.98 (1.28–3.06)
CXR_LP_	77/3,622 (2.1)	0.43 (0.26–0.71)	1.22 (0.97–1.55)	96/3,622 (2.7)	0.55 (0.34–0.88)	1.09 (0.88–1.34)
NLST						
First-round negative CXR						
CXR_SN_	404/19,359 (2.1)	1 (ref)	1 (ref)	690/19,359 (3.6)	1 (ref)	1 (ref)
CXR_GP_	74/881 (8.4)	4.03 (3.08–5.28)	4.04 (3.08–5.29)	50/881 (6.1)	1.45 (1.05–2.01)	1.45 (1.05–2.01)
First-round positive CXR						
CXR_SP_	19/169 (11.2)	1 (ref)	5.38 (3.38–8.56)	19/169 (11.2)	1 (ref)	3.35 (2.11–5.30)
CXR_LP_	35/1,585 (2.2)	0.16 (0.09–0.29)	0.89 (0.61–1.31)	64/1,585 (7.8)	0.29 (0.17–0.50)	0.99 (0.75–1.31)
Meta-analysis *						
First-round negative CXR						
CXR_SN_	1,073/65,187 (1.6)	1 (ref)	1 (ref)	1,654/65,187 (2.5)	1 (ref)	1 (ref)
CXR_GP_	197/3,462 (5.7)	3.33 (2.35–4.71)	2.88 (1.50–5.52)	155/3,462 (4.5)	1.68 (1.41–2.01)	1.58 (1.33–1.87)
First-round positive CXR						
CXR_SP_	39/624 (6.3)	1 (ref)	3.86 (2.03–7.35)	40/624 (6.4)	1 (ref)	2.56 (1.53–4.29)
CXR_LP_	112/5,207 (2.2)	0.27 (0.10–0.70)	1.08 (0.80–1.46)	163/5,207 (3.1)	0.40 (0.22–0.76)	1.05 (0.89–1.25)

CI, confidence interval; HR, hazard ratio; PLCO, Prostate, Lung, Colorectal, and Ovarian Cancer Screening Trial; NLST, National Lung Screening Trial.

a Multivariable Cox regression model adjusting for age, sex, race, education levels, marital status, smoking status, family history of lung cancer, BMI;

b Trajectories were combined as a single variable, and all variables listed in model 1 were adjusted in model 2.*Random-effects model was used.

Moreover, after incorporating the four CXR trajectories into a single variable, compared to CXR_SN_, the adjusted HR (95%CIs) of LC incidence based on meta-analyses were 2.88(1.50–5.52), 3.86(2.03–7.35), and 1.08(0.80–1.46) for CXR_GP_, CXR_SP_, and CXR_LP_, respectively, while adjusted HR (95%CIs) of LC mortality were 1.58(1.33–1.87), 2.56(1.53–4.29) and 1.05(0.89–1.25) (**Table 1**).

### 3.3 Association of CXR trajectories with LC risk by smoking status

In the PLCO trial, after stratifying by smoking status and adjusting other available baseline factors (**Table 2**), compared to CXR_SN_, CXR_GP_ was still significantly associated with increased LC incidence across different subgroups [HR (95%CIs): 4.94(2.79–8.73), 2.94(2.24–3.86) and 2.34(1.71–3.21) in never, sever, and current smokers] and associated with increased LC mortality in both ever and current smokers [1.88(1.43–2.47) and 1.43(1.03–1.97)] but not in never smokers [1.16(0.51–2.65)]. Compared to CXR_SP_, CXR_LP_ was significantly associated with decreased risks of both LC incidence and mortality in both never and ever smokers [0.24(0.07–0.82) and 0.38(0.18–0.80) for LC incidence, 0.41(0.13–1.29) and 0.40(0.21–0.78) for LC mortality] but not in current smokers [0.60 (0.25–1.42) for LC incidence and 0.96(0.39–2.35) for LC mortality] (**Table 2**).

**Table 2:** Stratified association of CXR trajectories with lung cancer and mortality by smoking status in the PLCO trial.

CXR trajectories	Lung cancer incidence			Lung cancer mortality		
	Events/Total (%)	Model 1 ^a^ HR (95% CI)	Model 2 ^b^ HR (95%CI)	Events/Total (%)	Model 1 ^a^ HR (95% CI)	Model 2 ^b^ HR (95%CI)
Never smokers						
First-round negative CXR						
CXR_SN_	59/22,874 (0.3)	1 (ref)	1 (ref)	100/22,874 (0.4)	1 (ref)	1 (ref)
CXR_GP_	15/1,091 (1.4)	4.94 (2.79-8.73)	4.89 (2.77-8.65)	6/1,091 (0.5)	1.16 (0.51-2.65)	1.16 (0.51-2.65)
First-round positive CXR						
CXR_SP_	4/224 (1.8)	1 (ref)	7.74 (2.81-21.37)	4/224 (1.8)	1 (ref)	4.61 (1.70-12.55)
CXR_LP_	8/1,635 (0.5)	0.24 (0.07-0.82)	1.69 (0.80-3.53)	13/1,635 (0.8)	0.41 (0.13-1.29)	1.62 (0.91-2.90)
Ever smokers						
First-round negative CXR						
CXR_SN_	357/19,065 (1.9)	1 (ref)	1 (ref)	520/19,065 (2.7)	1 (ref)	1 (ref)
CXR_GP_	61/1,155 (5.3)	2.94 (2.24-3.86)	2.95 (2.25-3.88)	47/1,155 (4.9)	1.88 (1.43-2.47)	1.89 (1.43-2.48)
First-round positive CXR						
CXR_SP_	9/180 (5.0)	1 (ref)	2.59 (1.33-5.02)	11/180 (6.1)	1 (ref)	2.20 (1.21-4.01)
CXR_LP_	33/1,637 (2.0)	0.38 (0.18-0.80)	0.95 (0.67-1.36)	44/1,637 (2.7)	0.40 (0.21-0.78)	0.92 (0.67-1.25)
Current smokers						
First-round negative CXR						
CXR_SN_	252/3,827 (6.6)	1 (ref)	1 (ref)	341/3,827 (8.9)	1 (ref)	1 (ref)
CXR_GP_	47/329 (14.3)	2.34 (1.71-3.21)	2.33 (1.70-3.18)	42/329 (12.8)	1.43 (1.03-1.97)	1.42 (1.03-1.97)
First-round positive CXR						
CXR_SP_	7/51 (13.7)	1 (ref)	2.26 (1.06-4.83)	6/51 (11.8)	1 (ref)	1.27 (0.57-2.87)
CXR_LP_	36/347 (10.4)	0.60 (0.25-1.42)	1.53 (1.08-2.17)	39/347 (11.2)	0.96 (0.39-2.35)	1.20 (0.86-1.68)

CI: Confidence Interval; HR: Hazard Ratio; PLCO: Prostate, Lung, Colorectal, and Ovarian Cancer Screening Trial.

**a:** multi-variable cox regression model adjusting for age, sex, race, education levels, marital status, family history of lung cancer, BMI;

**b:** CXR trajectories within the same smoking status were incorporated as a single variable, and all variables listed in model 1 were adjusted in model 2.

After incorporating the four CXR trajectories into a single variable, among never smokers, compared to CXR_SN_, higher risks of LC incidence and LC mortality were observed in CXR_SP_ than CXR_GP_ [HRs (95%CIs): 7.74(2.81–21.37) *vs*. 4.61(1.70–12.55)], and no significant differences in LC incidence and LC mortality were observed between CXR_SN_ and CXR_LP_. Similar trends were observed among ever and current smokers. However, higher risks of LC incidence were observed in CXR_GP_ than CXR_SP_ among both ever smokers [HRs (95%CIs): 2.95(2.25–3.88) *vs*. 2.59(1.33–5.02)] and current smokers [2.33(1.70–3.18) *vs*. 2.26(1.06–4.83)]. Moreover, among current smokers, even regression to negative CXR after continuing positive CXR, an increased risk of LC was still observed, with HRs (95%CIs) of 1.53(1.08–2.17) (**Table 2**).

### 3.4 Interaction between CXR trajectories and smoking on LC risk

Among participants with first-round negative CXR, compared to CXR_SN_ among never smokers, even no progression in CXR, the adjusted HRs (95%CIs) of LC incidence were 7.39(5.60–9.75) for ever smokers and 31.45(23.58–41.95) for current smokers, while HRs (95%CIs) of LC mortality were 6.30(5.07–7.81) and 27.17(21.65–34.11). If participants gained positive CXR, LC incidence risk significantly climbed to 22.04(15.37–31.60) for ever smokers and 71.97(48.82–106.09) for current smokers, while LC mortality risk climbed to 11.90(8.58–16.50) and 38.92(27.04–56.02) (**Table 3**). Among participants with first-round positive CXR, compared to CXR_SP_ among never smokers, even no regression in CXR, the adjusted HRs (95%CIs) of LC incidence were 2.93(0.89–9.63) for ever smokers and 12.07(3.43–42.45) for current smokers, while HRs (95%CIs) of LC mortality were 3.72(1.18–11.75) and 9.22(2.55–33.32). If participants lost their positive CXR, LC incidence risk significantly decreased to 1.08(0.38–3.07) for ever smokers and 7.73(2.72–22.02) for current smokers, while LC mortality risk decreased to 1.51(0.54–4.22) and 8.71(3.08–24.67) (**Table 3**).

**Table 3:** Interaction between CXR trajectories and smoking status with lung cancer and mortality by smoking status in the PLCO trial.

CXR trajectories	Lung cancer incidence Lung cancer mortality					
	Events/Total (%)	Model 1 ^a^ HR (95% CI)	Model 2 ^b^ HR (95%CI)	Events/Total (%)	Model 1 ^a^ HR (95% CI)	Model 2 ^b^ HR (95%CI)
First-round negative CXR						
Never smokers						
CXR_SN_	59/22,874 (0.3)	1 (ref)	1 (ref)	100/22,874 (0.4)	1 (ref)	1 (ref)
CXR_GP_	15/1,091 (1.4)	5.14 (2.91–9.06)	5.12 (2.90–9.03)	6/1,091 (0.5)	1.23 (0.54–2.80)	1.23 (0.54–2.79)
Ever smokers						
CXR_SN_	357/19,065 (1.9)	7.39 (5.60–9.75)	7.47 (5.66–9.86)	520/19,065 (2.7)	6.30 (5.07–7.81)	6.36 (5.13–7.90)
CXR_GP_	61/1,155 (5.3)	22.04 (15.37–31.60)	22.23 (15.51–31.88)	47/1,155 (4.9)	11.90 (8.58–16.50)	12.03 (8.68–16.69)
Current smoking						
CXR_SN_	252/3,827 (6.6)	31.45 (23.58–41.95)	31.77 (23.84–42.36)	341/3,827 (8.9)	27.17 (21.65–34.11)	27.35 (21.80–34.31)
CXR_GP_	47/329 (14.3)	71.97 (48.82–106.09)	72.46 (49.18–106.76)	42/329 (12.8)	38.92 (27.04–56.02)	39.08 (27.16–56.23)
First-round positive CXR						
Never smokers						
CXR_SP_	4/224 (1.8)	1 (ref)	7.39 (2.68–20.36)	4/224 (1.8)	1 (ref)	4.31 (1.59–11.71)
CXR_LP_	8/1,635 (0.5)	0.24 (0.07–0.81)	1.74 (0.83–3.64)	13/1,635 (0.8)	0.40 (0.13–1.22)	1.71 (0.96–3.06)
Ever smokers						
CXR_SP_	9/180 (5.0)	2.93 (0.89–9.63)	19.80 (9.80–40.00)	11/180 (6.1)	3.72 (1.18–11.75)	14.26 (7.64–26.61)
CXR_LP_	33/1,637 (2.0)	1.08 (0.38–3.07)	7.06 (4.60–10.83)	44/1,637 (2.7)	1.51 (0.54–4.22)	5.77 (4.04–8.25)
Current smokers						
CXR_SP_	7/51 (13.7)	12.07 (3.43–42.45)	68.24 (31.00–150.18)	6/51 (11.8)	9.22 (2.55–33.32)	34.15 (14.93–78.15)
CXR_LP_	36/347 (10.4)	7.73 (2.72–22.02)	48.81 (32.13–74.13)	39/347 (11.2)	8.71 (3.08–24.67)	33.21 (22.86–48.23)

CI, confidence interval; HR, hazard ratio; PLCO, Prostate, Lung, Colorectal, and Ovarian Cancer Screening Trial.

a Multivariable Cox regression model adjusted for age, sex, race, education levels, marital status, family history of lung cancer, and BMI;

b Both four CXR trajectories and smoking status were incorporated as a single variable in model 1.

After combing all the participants, compared to CXR_SN_ among never smokers, the adjusted HRs (95%CIs) of LC incidence and mortality for CXR_SP_ among never smokers were 7.39(2.68–20.36) and 4.31(1.59–11.71). If never smokers lost their positive CXR, both the increased risks of LC incidence [1.74(0.83–3.64)] and mortality [1.71(0.96–3.06)] decreased to non-significant level. Similar trends were observed among both ever and current smokers. However, significant higher risks of LC incidence and mortality for CXR_SP_ were also observed for both ever and current smokers, especially for current smokers [68.24(31.00–150.18)]. Ever these smokers lose their positive CXR, the increased risks of LC incidence and mortality did not decrease to non-significant level (**Table 3**).

### 3.5 Pathological characteristics of LC associated with CXR trajectories

As shown in **Table 4** and **Supplementary e-Tables S3,S4**, the proportion of advanced-stage LC in patients with CXR_GP_ was lower than those with CXR_SN_ [55.1% *vs*. 65.6% (p-value = 0.039) for PLCO, 39.2% *vs*. 66.3% (p-value < 0.001) for NLST, and 48.6% *vs*. 65.8% (p-value < 0.001) for meta-analysis], while there was no significant difference in cancer stages between CXR_SP_ and CXR_LP_. Similar lower proportion of LC with poor-grade differentiation were observed in participants with CXR_GP_ compared to those with CXR_SN_ [49.6% *vs*. 60.9% (p-value = 0.020) for meta-analysis], while no significant difference in cancer grades was observed between CXR_SP_ and CXR_LP_. Moreover, in the PLCO trial, non-significant differences in the histological types of LC were observed between CXR_SN_ and CXR_GP_, or between CXR_SP_ and CXR_LP_ (both p-value > 0.05, **Supplementary e-Table S3**).

**Table 4:** Association of CXR trajectories with pathological characteristics of lung cancer in the PLCO and NLST trials.

CXR trajectories	Stage			Grade		
	Early stage (I and II) ^a^	Advanced stage (III and IV) ^b^	*p*	High-grade differentiation ^c^	Poor-grade differentiation ^d^	*p*
PLCO						
First-round negative CXR			0.039			0.170
CXR_SN_	197(34.4)	375(65.6)		142(40.6)	208(59.4)	
CXR_GP_	48(44.9)	59(55.1)		41(48.8)	43(51.2)	
First-round positive CXR			0.946			1.000
CXR_SP_	7(36.8)	12(63.2)		7(50.0)	7(50.0)	
CXR_LP_	23(37.7)	38(62.3)		23(50.0)	23(50.0)	
**NLST**						
First-round negative CXR			<0.001			0.030
CXR_SN_	136(33.7)	267(66.3)		83(36.9)	142(63.1)	
CXR_GP_	45(60.8)	29(39.2)		30(52.6)	27(47.4)	
First-round positive CXR			0.095			0.238
CXR_SP_	4(22.2)	14(77.8)		6(66.7)	3(33.3)	
CXR_LP_	16(45.7)	19(54.3)		10(43.5)	13(56.5)	
Meta-analysis *						
First-round negative CXR			<0.001			0.020
CXR_SN_	333(34.2)	642(65.8)		225(39.1)	350(60.9)	
CXR_GP_	93(51.4)	88(48.6)		71(50.4)	70(49.6)	
First-round positive CXR			0.336			0.630
CXR_SP_	11(29.7)	26(70.3)		13(56.5)	10(43.5)	
CXR_LP_	39(40.6)	57(59.4)		33(47.8)	36(52.2)	

PLCO, Prostate, Lung, Colorectal, and Ovarian Cancer Screening Trial; NLST, National Lung Screening Trial.

*Random-effects model was used.

### 3.6 Potential factors associated with progression and regression in CXR

As shown in **Table 5**, in the PLCO trial, based on the multivariable logistic regression, progression in CXR (namely, CXR_GP_) was significantly associated with elder age [OR (95%CIs) for 70–74 years *vs*. 55–59 years: 1.58(1.39–1.79), p-value < 0.001], men [1.10(1.00–1.19), p-value = 0.042], widowed/divorced/separated status [1.19(1.07–1.31), p-value=0.001], and current smoking [1.79(1.59–2.04), p-value < 0.001]. However, regression in CXR (namely, CXR_LP_) was not significantly associated with any currently available baseline variables (**Table 5**). Similar associations were observed in the NLST trial (**Supplementary e-Table S5**).

**Table 5:** Factors associated with CXR trajectories in the PLCO trial.

Characteristics	CXR_GP_		CXR_LP_	
	OR (95% CI) ^a^	*p*	OR (95% CI) ^a^	*p*
Age (years)				
55–59	1 (ref)		1 (ref)	
60–64	1.10 (0.99–1.22)	0.074	0.88 (0.68–1.14)	0.328
65–69	1.25 (1.12–1.40)	<0.001	0.95 (0.73–1.25)	0.730
70–74	1.58 (1.39–1.79)	<0.001	1.06 (0.77–1.45)	0.723
Sex				
Women	1 (ref)		1 (ref)	
Men	1.10 (1.00–1.19)	0.042	1.03 (0.84–1.28)	0.765
Race				
White	1 (ref)		1 (ref)	
Other	1.08 (0.95–1.23)	0.223	0.78 (0.56–1.07)	0.120
Education levels				
<High school	1 (ref)		1 (ref)	
Senior high school	0.91 (0.77–1.06)	0.223	1.05 (0.71–1.56)	0.816
College or above	0.89 (0.76–1.05)	0.159	1.12 (0.76–1.64)	0.576
Marital status				
Married/Living as married	1 (ref)		1 (ref)	
Widowed/Divorced/Separated	1.19 (1.07–1.31)	0.001	0.87 (0.68–1.21)	0.286
Never Married	1.00 (0.80–1.27)	0.972	0.91 (0.52–1.58)	0.731
BMI (kg/m^2^)				
<18.5	1 (ref)		1 (ref)	
18.5–25	0.83 (0.53–1.30)	0.403	1.99 (0.84–4.70)	0.117
25–30	0.72 (0.46–1.12)	0.145	2.21 (0.93–5.22)	0.072
>30	0.75 (0.48–1.19)	0.223	1.87 (0.78–4.48)	0.158
Smoking status				
Never smoking	1 (ref)		1 (ref)	
Ever smoking	1.26 (1.16–1.38)	<0.001	1.23 (0.99–1.52)	0.064
Current smoking	1.79 (1.59–2.04)	<0.001	0.97 (0.70–1.36)	0.868
Family history of lung cancer				
No	1 (ref)		1 (ref)	
Yes	1.05 (0.93–1.20)	0.443	0.96 (0.70–1.30)	0.778

BMI, body mass index; CI, confidence interval; OR, odds ratio; PLCO, Prostate, Lung, Colorectal, and Ovarian Cancer Screening Trial.

a Multivariable logistic regression model adjusting for age, sex, race, education levels, marital status, smoking status, family history of lung cancer, and BMI.

## 4 Discussion

After systematically searching the comparative studies on the association between progression (or trajectory) on chest X-ray and LC risk published before 2023, unfortunately, we found few studies focused on this topic, while fewer studies explored the stratified association by smoking status. To the best of our knowledge, this was the first study to investigate the associations of CXR trajectories with LC risk, and this was also the first study to evaluate the interaction between CXR trajectories and smoking status on LC risk. Based on the two independent, well-curated, multicenter community-based LC screening trials, we discovered that LC risk varied across different CXR trajectories. Overall, progression in CXR was significantly associated with increased risk of LC incidence and mortality even after stratification according to smoking status, while regression in CXR was associated with decreased LC risk among never or ever smokers but not current smokers. Furthermore, significantly higher LC risks were observed in smokers than never smokers, even in the absence of obvious chest symptoms in CXR. Additionally, smoking was significantly associated with increased risk of progression in CXR but not with regression in CXR.

Previous studies suggested that chest X-ray screening for LC may have a false-negative rate of at least 20% (8), and the UK biobank study also supported that CXR failed to identify nearly 17.7% of lung cancer patients in the year before diagnosis (16). Similarly, CXR screening is also likely to have a high percentage of false positive (17–19). All these studies remind the general practitioners that a single round of CXR cannot rule out the risk of lung cancer; therefore, multiple rounds of CXR screening or monitoring are necessarily needed to reduce the missed or false diagnosis of LC (20). However, no studies had explored whether there was a significant difference in LC risk across different CXR trajectories, especially between CXR_SP_ and CXR_GP_. In this study, overall, LC risk with CXR_SP_ was significantly higher than that with CXR_GP_. This is easy to understand, since people with CXR_SP_ are likely to have a more serious chest disorder than those with CXR_GP_. This association can be more clearly observed in never smokers. However, this result cannot always be observed in any settings. Conversely, among either ever or current smokers, consistent higher risks of LC incidence were observed in CXR_GP_ than CXR_SP_. These results were likely to suggest that smoking-related CXR_GP_ may be associated with a more serious LC risk than smoking-related CXR_SP_, and this type of CXR_SP_ should deserve more attentions than CXR_GP_ in the never smokers.

The harmful effects of smoking have been fully addressed in the previous and latest WHO reports on the global tobacco epidemic 2021 (21), the previous and updated reports of the Surgeon General on the Health Consequences of Smoking (22), and the latest Cancer Atlas (third edition) released in 2019 (23). In summary, all smoked and traditional smokeless tobacco products cause cancer. Although lung cancer is the most dominated cancer caused by cigarette smoking, at least 19 other sites or sub-sites cancer are causally associated with smoking. Additionally, smoked tobacco products cause even more deaths from vascular and respiratory conditions than from cancer.

Consistent with a large body of previous research evidence (24–27), smoking can lead to an increased LC risk dozens of times, especially among current smokers. However, most previous studies suggest that smoking increases the LC risk primarily by increasing lung symptoms. In this study, we observed that even in people with stable negative CXR, smoking still increased the LC risk 7–30 times. These results suggest that smoking could increase the LC risk through other ways with no obvious symptoms in the lung. Although this was also reported in previous studies, it was rarely observed (28–30). On the other hand, although we observed a clear interaction between smoking and trajectories on LC risk, the increased risk of LC still existed among current smokers even if positive CXR regressed to negative CXR. Both evidence from CXR_SN_ and CXR_LP_ further suggested that smoking increased the LC risk not only by increasing lung symptoms but also by other ways without obvious lung symptoms. This was the second key finding of this study. This result suggested that we should not only focus on the obvious lung symptoms associated with smoking should but also learn more about how smoking increased the LC risk through other ways without obvious lung symptoms (20). The latter is frequently ignored in current LC prevention practice but would be very helpful in understanding the carcinogenic mechanisms of smoking.

Additionally, two other findings also deserved more attentions. First, as mentioned above, it was easy to understand that smoking was associated with increased risk of progression in CXR. This was consistent with several previous studies (24, 31). However, unexpectedly, we found no association between smoking and regression in CXR. This result may be related to the small sample size or the limited analytical variables available in this study. Several factors, such as diet, exercise, and previous lung diseases, may influence the regression in CXR. Lack of these information would bias the current results. The current results further supported the interaction between CXR trajectories and smoking on LC risk. However, the causes for regression in CXR may be much more complex than expected, and more research is needed to investigate these causes of CXR regression in the future. Second, among participants with first-round negative CXR, lower proportion of advanced LC and poorly differentiated LC were observed in CXR_GP_ than CXR_SN_. Since both advanced stage and poor differentiation are associated with worse prognosis, hence, participants with CXR_GP_ deserved special attention and more follow-up. This will allow us to detect potential LC earlier and to reduce the progression in CXR with effective interventions.

In addition to the interesting findings mentioned above, several limitations of this study should also be considered. First, the NLST trial did not include never smokers, and the rounds of CXR differed between the PLCO and NLST trials. Therefore, the current results would be biased by the potential heterogeneity between the PLCO and NLST trials. As noted above, the heterogeneity indeed existed. However, the overall association between CXR trajectories and LC risk from the two trials was very similar. Since the PLCO and NLST trials recruited two completely different populations, the results of the two cohorts can be seen as independently mutual validation. Moreover, meta-analyses with random-effects model were used to combine the results of the two cohorts; a more conservative conclusion was drawn in this study. Second, ignorance of the disordered fluctuations in CXR would bias the current results. In fact, the sample size of these populations was relatively small, and the clinical significance of these disorder fluctuations in CXR is unclear. Therefore, ignoring the disordered fluctuations would not have a significant impact on current results. Third, due to lack of never smokers in the NLST trial, stratified analyses by smoking and interaction analyses between CXR trajectories and smoking can only be conducted in the PLCO trial. This would also bias the current results. Further studies are needed to validate the current results. Fourth, CXR can only reflect relatively limited pulmonary symptoms; several minor progressions in CXR would be missed. LDCT trajectories or more detailed symptoms trajectories in the lung are needed to be investigated to detect potential LC earlier in the future.

## 5 Conclusion

Based on the two large-scale lung cancer screening trials, generally, progression in CXR was associated with increased LC risk, while regression was associated with decreased LC risk. However, smoking would greatly increase the LC risk even in the absence of obvious chest symptoms in CXR, which deserves more attention to explore the carcinogenic mechanisms of smoking. Moreover, smoking would reverse the higher LC risk with CXR_SP_ than CXR_GP_, which was observed in general participants or never smokers, and regression in CXR can only reverse the LC risk with positive CXR to the reference level as CXR_SN_ among never or ever smokers but not current smokers. Given the high incidence and mortality of LC worldwide, the high prevalence of smoking, and the great role of CXR trajectories with LC risk, comprehensive intervention incorporating CXR trajectories and smoking status should be recommended to reduce LC risk, especially in resource-limited regions, thereby preventing more life-threatening adverse clinical outcomes.

## Data availability statement

The original contributions presented in the study are included in the article/**Supplementary Material**. Further inquiries can be directed to the corresponding authors.

## Ethics statement

The studies involving human participants were reviewed and approved by The National Cancer Institute and their local institutional review board. The patients/participants provided their written informed consent to participate in this study.

## Author contributions

YL: conceptualization, methodology, data curation, formal analysis, investigation, writing—original draft, and writing—review and editing. ZWF: conceptualization, data curation, investigation, and writing—review and editing. ZYF: conceptualization, data curation, investigation, and writing—review and editing. YZ: conceptualization, data curation, investigation, and writing—review and editing. CL: conceptualization, methodology, data curation, and writing—review and editing. XL: conceptualization, data curation, investigation, and writing—review and editing. HD: conceptualization, data curation, investigation, and writing—review and editing. XC: conceptualization, investigation, and writing—review and editing. LZ: conceptualization, investigation, and writing—review and editing. CS: conceptualization, methodology, data curation, and writing—review and editing. LY: conceptualization, data curation, and writing—review and editing. YG: conceptualization, and writing—review and editing. XW: conceptualization, and writing—review and editing. QZ: conceptualization and writing—review and editing. ZL: conceptualization, data curation, and writing—review and editing. FFS: conceptualization, data curation, and writing—review and editing. YH: conceptualization, data curation, investigation, writing—original draft, writing—review and editing, and supervision. FJS: conceptualization, data curation, investigation, writing—review and editing, supervision, and funding acquisition.
