# Supplementary material for: Associations of chest X-ray trajectories, smoking, and the risk of lung cancer in two population-based cohort studies
Source: Front Oncol. 2023 Jul 18;13:1203320. doi: 10.3389/fonc.2023.1203320 (PMC10392917; doi:10.3389/fonc.2023.1203320)
Supplement: Supplementary file 1 [file DataSheet_1.docx]

**Supplementary documents

e-Figure 1. Flow chart of participants’ selection in the PLCO study.
e-Figure 2. Flow chart of participants’ selection in the NLST study.**

**e-Table 1. Baseline characteristics of different subgroups in the PLCO study.
e-Table 2. Baseline characteristics of different subgroups in the NLST study.
e-Table 3. Comparisons of pathological characteristics of lung cancers between different groups in the PLCO study.
e-Table 4. Comparisons of pathological characteristics of lung cancers between different groups in the NLST study.
e-Table 5. Factors associated with CXR trajectories in the NLST study.**

**
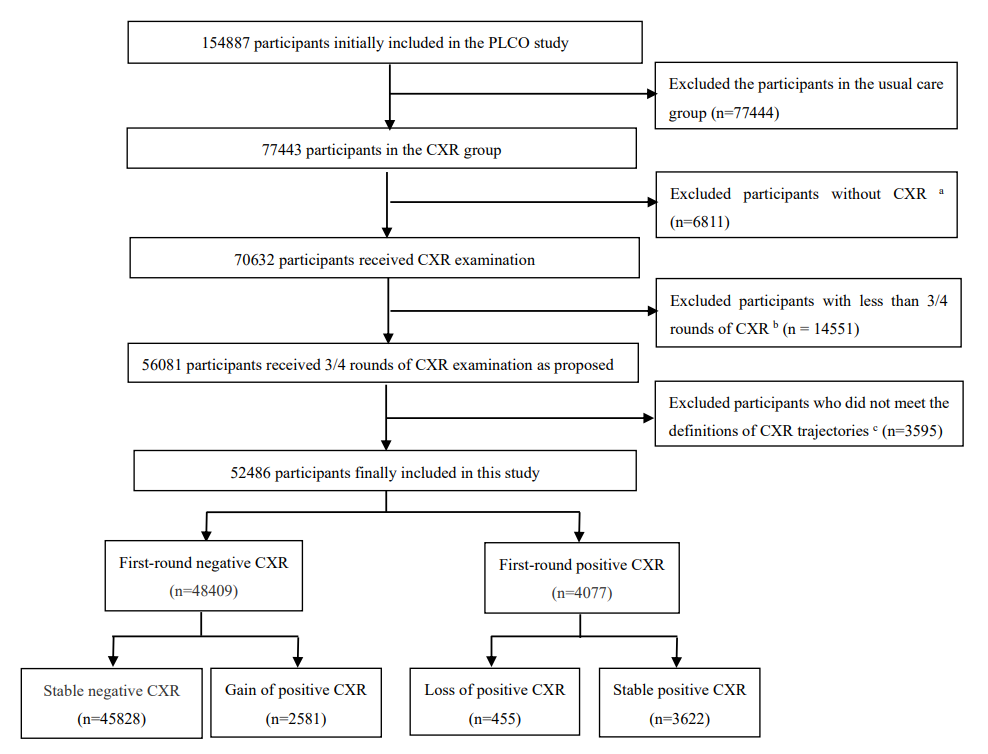
**


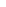


**e-Figure 1. Flow chart of screening participants’ selection in the PLCO study.**

**Note:** CXR: chest X-rays; PLCO: Prostate, Lung, Colorectal, and Ovarian Cancer Screening Trial.

**a:** including participants who did not receive any CXR examination and those without inadequate screen;
**b:** including participants with one round of CXR examination (n=3819), participants with two rounds of CXR examination (n=4937), participants with three rounds of CXR examination enrolled in 1993-1995 (n=2620), smoking participants with three rounds of CXR examination enrolled in 1996-2001 (n=3175).

**
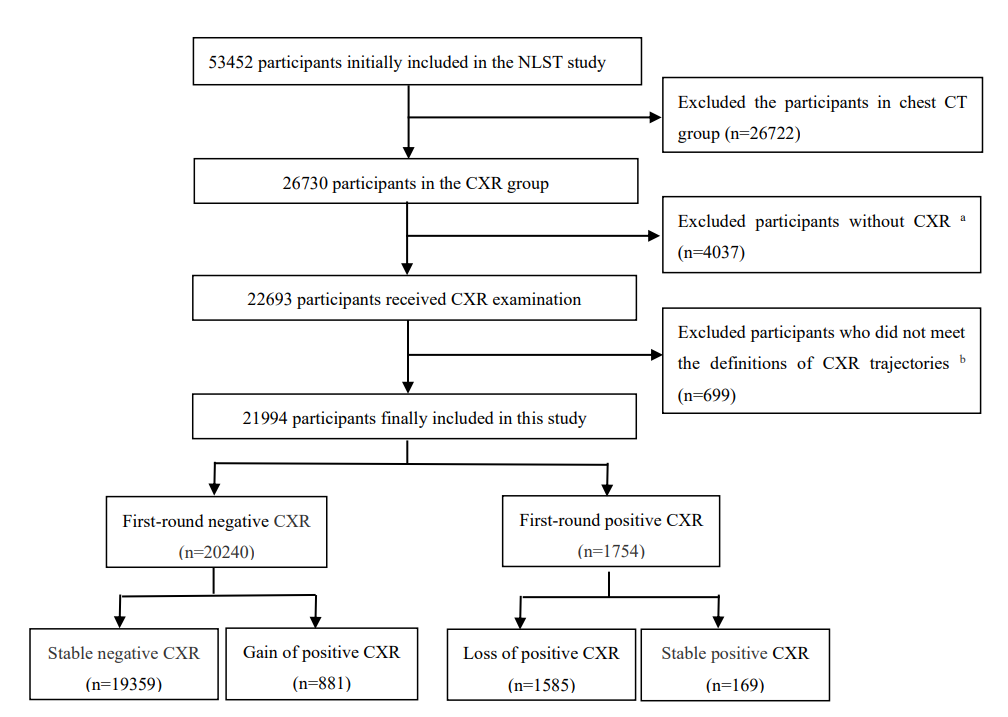
**


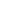


**e-Figure 2. Flow chart of participants’ selection in the NLST study.**

**Note:** CT: computed tomography; CXR: chest X-rays; NLST: National Lung Screening Trial.

**a:** including participants who did not receive any CXR examination and those without inadequate screen.

**e-Table 1. Baseline characteristics of different subgroups in the PLCO study.**

| **Characteristics** | **All**  **(N =52486)** | **First-round negative CXR** | | |  | **First-round positive CXR** | | |
| --- | --- | --- | --- | --- | --- | --- | --- | --- |
|  |  | **CXR_SN_**  **(N=45828)** | **CXR_GP_**  **(N=2581)** | ***P*** _value_ **^a^** |  | **CXR_SP_**  **(N=455)** | **CXR_LP_**  **(N=3622)** | ***P*** _value_ **^a^** |
| Age (years) |  |  |  | <0.001 |  |  |  | 0.636 |
| 55-59 | 18307(34.9) | 16318(35.6) | 802(31.1) |  |  | 128(28.1) | 1059(29.2) |  |
| 60-64 | 16288(31.0) | 14293(31.2) | 767(29.7) |  |  | 148(32.5) | 1080(29.8) |  |
| 65-69 | 11538(22.0) | 9931(21.7) | 608(23.6) |  |  | 111(24.4) | 888(24.5) |  |
| 70-74 | 6353(12.1) | 5286(11.5) | 404(15.7) |  |  | 68(14.9) | 595(16.4) |  |
| Sex |  |  |  | 0.015 |  |  |  | 0.212 |
| Women | 25626(48.8) | 22609(49.3) | 1210(46.9) |  |  | 213(46.8) | 1584(43.7) |  |
| Men | 26870(51.2) | 23219(50.7) | 1371(53.1) |  |  | 242(53.2) | 2038(56.3) |  |
| Race |  |  |  | 0.047 |  |  |  | 0.120 |
| White | 47116(89.8) | 41119(89.9) | 2282(88.7) |  |  | 404(88.8) | 3311(91.4) |  |
| Other | 5290(10.1) | 4640(10.1) | 292(11.3) |  |  | 51(11.2) | 307(8.6) |  |
| Education levels |  |  |  | 0.001 |  |  |  | 0.575 |
| < Senior high school | 3337(6.4) | 2851(6.2) | 197(7.7) |  |  | 35(7.7) | 254(7.0) |  |
| Senior high school | 18368(35.1) | 16058(35.1) | 901(35.1) |  |  | 163(35.8) | 1246(34.5) |  |
| College or above | 30664(58.5) | 26824(58.7) | 1471(57.2) |  |  | 257(56.5) | 2112(58,5) |  |
| Marital status |  |  |  | <0.001 |  |  |  | 0.324 |
| Married/Living as married | 41072(78.4) | 35962(78.7) | 1933(75.2) |  |  | 343(75.4) | 2834(78.4) |  |
| Widowed/Divorced/Separated | 9616(18.4) | 8296(18.1) | 558(21.7) |  |  | 97(21.3) | 665(18.4) |  |
| Never Married | 1687(3.2) | 1478(3.2) | 80(3.1) |  |  | 15(3.3) | 114(3.2) |  |
| BMI (kg/m^2^) |  |  |  | 0.003 |  |  |  | 0.103 |
| <18.5 | 321(0.6) | 268(0.6) | 21(0.8) |  |  | 7() | 25(0.7) |  |
| 18.5-25 | 17079(32.9) | 14825(32.7) | 903(35.5) |  |  | 154() | 1197(33.4) |  |
| 25-30 | 22331(43.0) | 19544(43.1) | 1037(40.8) |  |  | 178() | 1572(43.9) |  |
| ≥30 | 12207(23.5) | 10728(23.6) | 583(22.9) |  |  | 108() | 788(22.0) |  |
| Smoking status |  |  |  | <0.001 |  |  |  | 0.120 |
| Never smoking | 25824(49.2) | 22874(50.0) | 1091(42.4) |  |  | 224(49.2) | 1635(45.1) |  |
| Former smoking | 22037(42.0) | 19065(41.7) | 1155(44.9) |  |  | 180(39.6) | 1637(45.2) |  |
| Current smoking | 4554(8.7) | 3827(8.3) | 329(12.7) |  |  | 51(11.2) | 347(9.6) |  |
| Family history of lung cancer |  |  |  | 0.154 |  |  |  | 0.522 |
| No | 45209(89.2) | 39545(89.3) | 2198(88.8) |  |  | 381(88.0) | 3085(88.5) |  |
| Yes | 5454(10.8) | 4723(10.7) | 279(11.2) |  |  | 52(12.0) | 400(11.5) |  |

**Note:** BMI: body mass index; PLCO: Prostate, Lung, Colorectal, and Ovarian Cancer Screening Trial.

**a:** *P* _values_ from the χ2 test.

**e-Table 2. Baseline characteristics of different subgroups in the NLST study.**

| **Characteristics** | **All**  **(N =21994)** | **First-round negative CXR** | | |  | **First-round positive CXR** | | |
| --- | --- | --- | --- | --- | --- | --- | --- | --- |
|  |  | **CXR_SN_**  **(N=19359)** | **CXR_GP_**  **(N=881)** | ***P*** _value_ **^a^** |  | **CXR_SP_**  **(N=169)** | **CXR_LP_**  **(N=1585)** | ***P*** _value_ **^a^** |
| Age (years) |  |  |  | <0.001 |  |  |  | 0.011 |
| 55-59 | 9431(42.9) | 8436(43.6) | 343(38.9) |  |  | 45(26.6) | 607(38.3) |  |
| 60-64 | 6780(30.8) | 5971(30.8) | 256(29.1) |  |  | 68(40.2) | 485(30.6) |  |
| 65-69 | 3912(17.8) | 3382(17.5) | 185(21.0) |  |  | 32(18.9) | 313(19.7) |  |
| 70-74 | 1871(8.5) | 1570(8.1) | 97(11.0) |  |  | 24(14.2) | 180(11.4) |  |
| Sex |  |  |  | 0.574 |  |  |  | 0.057 |
| Women | 8901(40.5) | 7919(40.9) | 352(40.0) |  |  | 72(42.6) | 558(35.2) |  |
| Men | 13093(59.5) | 11440(59.1) | 529(60.0) |  |  | 97(57.4) | 1027(64.8) |  |
| Race |  |  |  | 0.539 |  |  |  | 0.036 |
| White | 20175(91.7) | 17752(91.7) | 813(92.3) |  |  | 148(87.6) | 1462(92.2) |  |
| Other | 1819(8.3) | 1607(8.3) | 68(7.7) |  |  | 21(12.4) | 123(7.8) |  |
| Education levels |  |  |  | 0.086 |  |  |  | 0.548 |
| < Senior high school | 1206(6.6) | 1040(6.4) | 58(7.9) |  |  | 13(9.1) | 95(7.3) |  |
| Senior high school | 8301(45.2) | 7288(45.1) | 345(47.2) |  |  | 69(48.3) | 599(45.9) |  |
| College or above | 8840(48.2) | 7840(48.5) | 328(44.9) |  |  | 61(42.7) | 611(46.8) |  |
| Marital status |  |  |  | 0.362 |  |  |  | 0.511 |
| Married/Living as married | 15099(68.9) | 13257(68.7) | 602(68.7) |  |  | 123(73.2) | 1117(70.6) |  |
| Widowed/Divorced/Separated | 5862(26.7) | 5149(26.9) | 227(25.9) |  |  | 41(24.4) | 400(25.3) |  |
| Never Married | 967(4.4) | 851(4.4) | 47(5.4) |  |  | 4(2.4) | 65(4.1) |  |
| BMI (kg/m^2^) |  |  |  | 0.139 |  |  |  | 0.003 |
| <18.5 | 206(0.9) | 167(0.9) | 13(1.5) |  |  | 6(3.6) | 20(1.3) |  |
| 18.5-25 | 6583(30.0) | 5744(29.8) | 271(30.8) |  |  | 64(37.9) | 504(31.9) |  |
| 25-30 | 9225(42.1) | 8118(42.1) | 375(42.7) |  |  | 74(43.8) | 658(41.6) |  |
| ≥30 | 5907(27.0) | 5262(27.3) | 220(25.0) |  |  | 25(14.8) | 400(25.3) |  |
| Smoking status |  |  |  | 0.804 |  |  |  | 0.331 |
| Former smoking | 11810(53.7) | 10399(53.7) | 477(54.1) |  |  | 84(49.7) | 850(53.6) |  |
| Current smoking | 10184(46.3) | 8960(46.3) | 404(45.9) |  |  | 85(50.3) | 735(46.4) |  |
| Family history of lung cancer |  |  |  | 0.807 |  |  |  | 0.930 |
| No | 17700(80.5) | 15581(80.5) | 712(80.8) |  |  | 136(80.5) | 1271(80.2) |  |
| Yes | 4294(19.5) | 3778(19.5) | 169(19.2) |  |  | 33(19.5) | 314(19.8) |  |

**Note:** BMI: body mass index; NLST: National Lung Screening Trial.

***:** *P* _values_ from the χ2 test.

**e-Table 3. Comparisons of pathological characteristics of lung cancers between different groups in the PLCO study.**

|  | **All**  **(N=889)** | **First-round negative CXR** | | |  | **First-round positive CXR** | | |
| --- | --- | --- | --- | --- | --- | --- | --- | --- |
| **Characteristics** |  | **CXR_SN_**  **(N=669)** | **CXR_GP_ (N=123)** | ***P*** |  | **CXR_SP_ (N=20)** | **CXR_LP_ (N=77)** | ***P*** |
| **Stage** |  |  |  |  |  |  |  |  |
| IA | 134(15.1) | 98(14.7) | 22(17.9) | 0.059 |  | 4(20.0) | 10(13.0) | 0.323 |
| IB | 89(10.0) | 60(9.0) | 19(15.4) |  |  | 2(10.0) | 8(10.4) |  |
| IIA | 11(1.2) | 7(1.1) | 2(1.6) |  |  | 0(0.0) | 2(2.6) |  |
| IIB | 41(4.6) | 32(4.8) | 5(4.1) |  |  | 1(5.0) | 3(3.9) |  |
| IIIA | 88(9.9) | 58(8.7) | 17(13.8) |  |  | 5(25.0) | 8(10.4) |  |
| IIIB | 93(10.5) | 72(10.8) | 13(10.6) |  |  | 3(15.0) | 5(6.5) |  |
| IV | 303(34.2) | 245(36.8) | 29(23.6) |  |  | 4(20.0) | 25(32.5) |  |
| Unknown | 127(14.3) | 94(14.1) | 16(13.0) |  |  | 1(5.0) | 16(20.8) |  |
| **Histology** |  |  |  |  |  |  |  |  |
| Small cell carcinoma | 127(14.3) | 94(14.0) | 16(13.0) | 0.768 |  | 1(5.0) | 16(20.8) | 0.098 |
| Non-small cell carcinoma | 762(85.7) | 577(86.0) | 107(87.0) |  |  | 19(95.0) | 61(79.2) |  |
| Small cell carcinoma | 127(14.3) | 94(14.0) | 16(13.0) | 0.903 |  | 1(5.0) | 16(20.8) | 0.110 |
| Adenocarcinoma | 303(34.1) | 233(34.7) | 45(36.6) |  |  | 3(15.0) | 22(28.6) |  |
| Squamous cell carcinoma | 170(19.1) | 125(18.6) | 20(16.3) |  |  | 8(40.0) | 17(22.1) |  |
| Other small cell carcinoma | 289(32.5) | 219(32.6) | 42(34.1) |  |  | 8(40.0) | 22(28.6) |  |
| **Grade** |  |  |  |  |  |  |  |  |
| Well differentiation | 60(6.7) | 39(5.8) | 13(10.6) | 0.008 |  | 1(5.0) | 7(9.1) | 0.686 |
| Moderately differentiation | 153(17.2) | 103(15.4) | 28(22.8) |  |  | 6(30.0) | 16(20.8) |  |
| Poorly differentiation | 237(26.7) | 179(26.7) | 36(29.3) |  |  | 6(30.0) | 16(20.8) |  |
| Undifferentiation | 44(4.9) | 29(4.3) | 7(5.7) |  |  | 1(5.0) | 7(9.1) |  |
| Unknown | 395(44.4) | 321(47.8) | 39(31.7) |  |  | 6(30.0) | 31(40.3) |  |

**Note:** PLCO: Prostate, Lung, Colorectal, and Ovarian Cancer Screening Trial.

**e-Table 4. Comparisons of pathological characteristics of lung cancers between different groups in the NLST study.**

|  | **All**  **(N =532)** | **First-round negative CXR** | | |  | **First-round positive CXR** | | |
| --- | --- | --- | --- | --- | --- | --- | --- | --- |
| **Characteristics** |  | **CXR_SN_**  **(N=404)** | **CXR_GP_**  **(N=74)** | ***P*** |  | **CXR_SP_**  **(N=19)** | **CXR_LP_**  **(N=35)** | ***P*** |
| **Stage** |  |  |  |  |  |  |  |  |
| IA | 102(19.2) | 65(16.1) | 26(35.1) | <0.001 |  | 2(10.5) | 9(25.7) | 0.128 |
| IB | 53(10.0) | 35(8.7) | 12(16.2) |  |  | 0(0.0) | 6(17.1) |  |
| IIA | 21(3.9) | 12(3.0) | 6(8.1) |  |  | 2(10.5) | 1(2.9) |  |
| IIB | 25(4.7) | 24(5.9) | 1(1.4) |  |  | 0(0.0) | 0(0.0) |  |
| IIIA | 57(107) | 43(10.6) | 11(14.9) |  |  | 2(10.5) | 1(2.9) |  |
| IIIB | 63(11.8) | 50(12.4) | 6(8.1) |  |  | 2(10.5) | 5(14.3) |  |
| IV | 209(39.3) | 174(43.1) | 12(16.2) |  |  | 10(52.6) | 13(37.1) |  |
| Unknown | 2(0.4) | 1(0.2) | 0(0.0) |  |  | 1(5.3) | 0(0.0) |  |
| **Grade** |  |  |  |  |  |  |  |  |
| Well differentiation | 26(4.9) | 15(3.7) | 7(9.5) | 0.001 |  | 3(15.8) | 1(2.9) | 0.213 |
| Moderately differentiation | 103(19.4) | 68(16.8) | 23(31.1) |  |  | 3(15.8) | 9(25.7) |  |
| Poorly differentiation | 157(29.5) | 117(29.0) | 25(33.8) |  |  | 3(15.8) | 12(34.3) |  |
| Undifferentiation | 28(5.3) | 25(6.2) | 2(2.7) |  |  | 0(0.0) | 1(2.9) |  |
| Unknown | 218(41.0) | 179(44.3) | 17(23.0) |  |  | 10(52.6) | 12(34.3) |  |

**Note:** NLST: National Lung Screening Trial.

**e-Table 5. Factors associated with CXR trajectories in the NLST study.**

| **Characteristics** | **CXR_GP_** | |  | **CXR_LP_** | |
| --- | --- | --- | --- | --- | --- |
|  | **OR (95% CI) ^a^** | ***P*** |  | **OR (95% CI) ^a^** | ***P*** |
| Age (years) |  |  |  |  |  |
| 55-59 | 1(ref) |  |  | 1(ref) |  |
| 60-64 | 1.02(0.85-1.22) | 0.842 |  | 0.52(0.34-0.81) | 0.003 |
| 65-69 | 1.24(1.01-1.53) | 0.040 |  | 0.75(0.44-1.28) | 0.291 |
| 70-74 | 1.48(1.15-1.92) | 0.003 |  | 0.59(0.33-1.08) | 0.086 |
| Sex |  |  |  |  |  |
| Women | 1(ref) |  |  | 1(ref) |  |
| Men | 1.06(0.90-1.24) | 0.495 |  | 1.45(0.99-2.10) | 0.051 |
| Race |  |  |  |  |  |
| White | 1(ref) |  |  | 1(ref) |  |
| Other | 0.94(0.71-1.23) | 0.644 |  | 0.53(0.31-0.92) | 0.024 |
| Education levels |  |  |  |  |  |
| < high school | 1(ref) |  |  | 1(ref) |  |
| Senior high school | 0.84(0.63-1.12) | 0.242 |  | 1.14(0.60-2.19) | 0.688 |
| College or above | 0.75(0.56-1.01) | 0.054 |  | 1.30(0.67-2.50) | 0.437 |
| Marital status |  |  |  |  |  |
| Married/Living as married | 1(ref) |  |  | 1(ref) |  |
| Widowed/Divorced/Separated | 1.00(0.84-1.19) | 0.968 |  | 1.43(0.92-2.23) | 0.109 |
| Never Married | 1.50(1.07-2.11) | 0.019 |  | 1.30(0.44-3.79) | 0.636 |
| BMI (kg/m^2^) |  |  |  |  |  |
| <18.5 | 1(ref) |  |  | 1(ref) |  |
| 18.5-25 | 0.58(0.31-1.09) | 0.090 |  | 2.12(0.79-5.68) | 0.137 |
| 25-30 | 0.54(0.29-1.01) | 0.055 |  | 2.35(0.87-6.36) | 0.092 |
| >30 | 0.52(0.28-0.99) | 0.046 |  | 4.71(1.61-13.71) | 0.005 |
| Smoking status |  |  |  |  |  |
| Former smoking | 1(ref) |  |  | 1(ref) |  |
| Current smoking | 0.92(0.79-1.07) | 0.289 |  | 0.80(0.55-1.15) | 0.220 |
| Family history of lung cancer |  |  |  |  |  |
| No | 1(ref) |  |  | 1(ref) |  |
| Yes | 0.97(0.81-1.18) | 0.781 |  | 1.06(0.69-1.63) | 0.804 |

**Note:** BMI: body mass index; CI: confidence interval; OR: Odd Ratio; NLST: National Lung Screening Trial.

**a:** multi-variable logistic regression model adjusting for age, sex, race, education levels, marital status, smoking status, family history of lung cancer, BMI.
